# P-SAMS: a web site for plant artificial microRNA and synthetic *trans*-acting small interfering RNA design

**DOI:** 10.1093/bioinformatics/btv534

**Published:** 2015-09-17

**Authors:** Noah Fahlgren, Steven T. Hill, James C. Carrington, Alberto Carbonell

**Affiliations:** Donald Danforth Plant Science Center, St. Louis, MO 63132, USA

## Abstract

**Summary:** The Plant Small RNA Maker Site (P-SAMS) is a web tool for the simple and automated design of artificial miRNAs (amiRNAs) and synthetic *trans*-acting small interfering RNAs (syn-tasiRNAs) for efficient and specific targeted gene silencing in plants. P-SAMS includes two applications, P-SAMS amiRNA Designer and P-SAMS syn-tasiRNA Designer. The navigation through both applications is wizard-assisted, and the job runtime is relatively short. Both applications output the sequence of designed small RNA(s), and the sequence of the two oligonucleotides required for cloning into ‘B/c’ compatible vectors.

**Availability and implementation:** The P-SAMS website is available at http://p-sams.carringtonlab.org.

**Contact:** acarbonell@ibmcp.upv.es or nfahlgren@danforthcenter.org

## 1 Introduction

Artificial miRNAs (amiRNAs) and synthetic *trans*-acting small interfering RNAs (syn-tasiRNAs) are two classes of designed small RNAs used to silence plant transcripts with high sequence complementarity (Ossowski *et al.*, 2008; Tiwari *et al.*, 2014; Zhang, 2014). AmiRNA and syn-tasiRNA differ in their biogenesis but are functionally similar. Methods to computationally design plant small RNAs are based on several criteria, including the requirement for a high degree of small RNA:target RNA base pairing (Ahmed *et al.*, 2015; Ossowski *et al.*, 2008; Schwab *et al.*, 2006).

We recently reported efficient molecular methods to generate amiRNA and syn-tasiRNA constructs by ligation of synthesized dsDNA oligonucleotides that include the amiRNA or syn-tasiRNA sequence into ‘B/c’ expression vectors (Carbonell *et al.*, 2014, 2015). B/c amiRNA vectors were tested in both monocots and eudicots and express a single amiRNA that targets one or multiple sequence-related transcripts. B/c syn-tasiRNA vectors express several syn-tasiRNAs targeting sequence-unrelated transcripts in *Arabidopsis* and closely related species.

P-SAMS is a wizard-assisted web tool for the simple and automated design of plant amiRNAs and syn-tasiRNAs. P-SAMS includes two applications, P-SAMS amiRNA Designer and P-SAMS syn-tasiRNA Designer. Both applications output a list of recommended amiRNA or syn-tasiRNA, and the sequence of the two oligonucleotides required for cloning into compatible B/c vectors.

## 2 Application description

### 2.1 Computational design of artificial small RNAs

The computational design of amiRNA and syn-tasiRNA is similar. All possible target sites are identified by cataloging the complete set of 21-nucleotide sequences from all input transcripts, including isoforms (foreground set). If off-target filtering is enabled, the foreground target site set is filtered to remove sites that contain a 15-nucleotide sequence from positions 6–20 (core target pairing sequence) that perfectly match a transcript that is not contained in the input set (background set). The remaining sites are grouped by the core target pairing sequence, and only target site groups that contain all input genes are considered further. Grouped sites are scored and ranked based on group-wise similarity and the identity of nucleotides at specific positions (positions 1, 2, 3 and 21). For each group of sites, a guide RNA is designed to target all sites with the additional criteria that (i) the guide RNA has a 5′U nucleotide, (ii) position 19 of the guide is a C and (iii) position 21 is intentionally mismatched. Finally, P-SAMS uses TargetFinder (Fahlgren and Carrington, 2010) to predict target RNAs for each guide RNA. Guide RNAs predicted to target exclusively transcripts from input gene set are output as ‘Optimal Results’; guide RNAs predicted to target transcripts from non-input genes are output as ‘Sub-optimal Results’. Up to three optimal and/or sub-optimal results are returned.

### 2.2 Website description and navigation

P-SAMS has a user-friendly interface and wizard-assisted navigation that guides the user during the design process. The wizard asks questions or requests information. To advance, questions are answered by clicking the button with the desired option, or the requested information is entered before clicking the ‘Next’ button. Help boxes pop out when ‘Help’ is clicked. The user can navigate back to the previous window by clicking the ‘Back’ button, or re-start the whole design process by clicking ‘Start Over’. When submitting a job, (i) warning messages alert the user to possible problems with the input information or combination of choices, and (ii) error messages are displayed if the user adds incorrect information. Median job time for single-targeting amiRNA design using P-SAMS was 2.45 and 3.22 min in *Arabidopsis thaliana* and *Oryza sativa*, respectively (*n* = 50 randomly selected target genes in both cases). Results include the sequence of the guide RNA and the sequence of the two oligonucleotides required for cloning in compatible B/c vectors. Also, for each designed guide RNA, a summary of TargetFinder results is displayed. In the P-SAMS syn-tasiRNA Designer results page, the user navigates to the ‘Build Construct’ page to build the syn-tasiRNA construct.

The ‘Frequently Asked Questions’ section includes links to the cloning protocol and to B/c vectors-related information, video tutorials and additional instructions. The ‘About’ section provides information regarding contact, author contributions, methods, software, how to cite, acknowledgements and the MIT License.

## 3 Implementation

P-SAMS website uses several modern libraries to interface with the user. The look-and-feel of the website was created using the CSS library Bootstrap 3 (www.getbootstrap.com). The front end was written with AngularJS (https://angularjs.org/), and the backend with a RESTful model on top of the Symfony2 (http://symfony.com/) PHP framework. Symfony acts as the middle layer between the P-SAMS job scheduler daemon and the web application front end. P-SAMS source code was written in Perl and uses MySQL (http://www.mysql.com/) to store the plant species databases. P-SAMS daemon is a Python script that coordinates JSON input/output between P-SAMS and Symfony web service. If the target specificity module is activated, P-SAMS runs TargetFinder v.1.7 (https://github.com/carringtonlab/TargetFinder) on a single CPU or in parallel using the Terascale Open-source Resource and QUEue Manager.
